# Molecular imaging of coronary atherosclerosis; predictive of an acute myocardial infarction?

**DOI:** 10.1007/s12471-013-0500-1

**Published:** 2013-11-28

**Authors:** E. E. van der Wall

**Affiliations:** Interuniversity Cardiology Institute of the Netherlands (ICIN) - Netherlands Heart Institute (NHI), P.O. Box 19258, 3501 DG Utrecht, the Netherlands

Coronary atherosclerosis is a leading cause of cardiovascular morbidity and mortality worldwide. Plaque complications occur most commonly from plaque rupture, and also from plaque erosion and calcified nodule formation. Over the past years, advanced structural, metabolic and molecular imaging technologies have emerged and offer new windows into atherosclerosis pathophysiology [1–4]. Molecular imaging complements traditional structural plaque imaging through the use of targeted probes that identify specific molecules and/or biological processes in vivo [5]. Preclinical atherosclerosis molecular imaging has successfully identified nearly all established high-risk plaque characteristics including inflammation, thrombosis, neo-vessel formation, apoptosis, and haemorrhage. However, clinical translation of molecular imaging has been slow compared with the rapid growth within the field.

Currently, clinical atherosclerosis molecular imaging is dominated by non-invasive PET metabolism/inflammation plaque imaging with fluorine 18 (18F)-fluorodeoxyglucose (FDG). For the past two decades, 18F FDG has been the only fluorine-18 radioligand approved by the US Food and Drug Administration (FDA) for PET imaging. Coronary FDG PET is challenging, in part owing to the small size of coronary vessels and cardiac motion artefacts, but also because of the intense FDG uptake of the adjacent highly metabolic myocardium that obscures the coronary FDG signal. However, with dietary myocardial suppression protocols, detection of FDG PET signal in the left main and proximal coronary artery beds is sometimes achievable. Recently, non-invasive coronary FDG PET activity has been investigated in the stented culprit lesions of 20 subjects with acute coronary syndromes (ACS) versus seven non-ACS subjects within 1 week of stent placement [6]. Using a predetermined FDG target to background ratio of 2.0 or greater to identify FDG-positive lesions, the ACS patients correlated positively with elevated stent FDG signal. However, coronary FDG signal was interpretable in only 50 % of ACS patients, implying the need for further improvements in coronary FDG PET.

Apart from 18F FDG coronary imaging, clinically available approaches such as sodium 18F-fluoride (NaF) for PET are emerging. Coronary calcification is a hallmark of advanced atheroma that can be detected with non-invasive NaF PET, which has been used for the past 3 decades to detect cancer bone metastases. As opposed to FDG, background myocardial uptake of NaF is negligible, and therefore NaF can determine coronary osteogenic activity. In a study by Dweck et al. from the University of Edinburgh [7], NaF PET molecular imaging identified sites of active coronary calcification non-invasively by PET and may be a new therapeutic target. The NaF signal was elevated in proportion to coronary CT calcium scores in 119 subjects, except in those with the highest calcium scores that may represent end-stage calcified plaques. From the same research group, Joshi et al. [8] recently (Lancet November 2013) studied coronary plaque imaging using two different radioactive tracers: 18F-NaF and 18F-FDG. The Scottish study included 40 patients with acute myocardial infarction (AMI) and 40 patients with stable angina. All patients underwent PET-CT, invasive coronary angiography, CT coronary angiography, and CT calcium scoring.

Among the 40 patients with AMI, 18F-NaF PET uptake was 34 % higher in the culprit ruptured plaque compared with non-culprit plaques. Nearly all patients (93 %) had increased 18F-NaF uptake in the culprit plaque. With 18F-FDG, however, no differences in uptake between culprit and non-culprit plaques were observed. Among the 40 patients with stable angina, 45 % had plaques with increased 18F-NaF uptake. These plaques were mostly non-obstructive at coronary angiography and contained more high-risk features on intravascular ultrasound compared with plaques without tracer uptake. Consequently, 18F-NaF PET may become an effective tool for predicting an AMI, which would have a great impact for patients with coronary atherosclerosis. Early detection of the atherosclerotic ‘danger zones’ could include immediate instalment of drugs such as statins or aspirin, drastic changes in lifestyle and even inserting stents into the affected coronary artery.

The use of 18F-NaF PET-CT offers the first non-invasive imaging method to identify and localise ruptured and high-risk coronary plaques. A next step is to show that increased 18F-NaF predicts future adverse clinical events. Further studies are therefore needed to evaluate whether the detection of risky plaques before rather than after an AMI has the potential to save lives. To summarise, the addition of newer molecular imaging tools such as NaF PET-CT will continue to strengthen our understanding of the in vivo biology of high-risk plaques [9, 10]. They are increasingly being translated into clinical use, and in combination with structural imaging they provide more comprehensive information to build risk-prediction tools. Ongoing efforts to improve non-invasive coronary imaging strategies must be pursued. Overall, with continued advances in molecular and structural atherosclerosis imaging, high-risk coronary atherosclerotic plaques will be brightened as never before. The recent study in the Lancet at least suggests that molecular imaging using PET-CT scanning may provide an answer in identifying ‘ticking time bomb’ patients at risk of an AMI.
